# Association of IL-10 Gene Polymorphisms and Human T Lymphotropic Virus Type I-Associated Myelopathy/tropical Spastic Paraparesis in North-East of Iran (Mashhad)

**Published:** 2013-03

**Authors:** Abbas Shirdel, Mahmoud Reza Azarpazhooh, Maryam Sahebari, Mohsen Ghanbari, Seyedeh Zahra Mirfeizi, Ian Hutchinson, Aghigh Ziaee, Houshang Rafatpanah

**Affiliations:** 1 Department of Hematology, School of Medicine, Mashhad University of Medical Sciences, Mashhad, Iran; 2 Neurology Department, School of Medicine, Mashhad University of Medical Sciences, Mashhad, Iran; 3 Rheumatic Diseases Research Centre, School of Medicine, Mashhad University of Medical Sciences, Mashhad, Iran; 4 Immunology Research Centre, School of Medicine, Mashhad University of Medical Sciences, Mashhad, Iran; 5 Rheumatic Diseases Research Centre, School of Medicine, Mashhad University of Medical Sciences, Mashhad, Iran; 6 Immunology Research Group, Faculty of Life Sciences, The University of Manchester, United Kingdom; 7 Inflammation and Inflammatory Disease Research Centre, School of Medicine, Mashhad University of Medical Sciences, Mashhad, Iran

**Keywords:** Gene, HAM/TSP, HTLV-I, IL-10, Polymorphisms

## Abstract

The underlying mechanisms leading to the development of human T-cell lymphotropic virus type I (HTLV-I) associated myelopathy/tropical spastic paraparesis (HAM/TSP) in HTLV-I infected individuals are not fully understood. Host genetic factors appear to be involved as risk factors for developing HAM/TSP. We investigated the possible contribution of interleukin-10 (IL-10) as a risk factor to HAM/TSP by comparing frequencies of promoter region single nucleotide polymorphisms in HTLV-I infected Iranian patients who either remained asymptomatic or developed HAM/TSP and asymptomatic HTLV-I carriers. Healthy, uninfected individuals from the same region served as healthy controls. Significant differences were observed in the distribution of IL-10 promoter alleles and genotypes at position -819 and -592 between HAM/TSP patients and healthy controls (*P=*0.01), and between HTLV-I carriers and healthy controls (*P=*0.02). The frequency of the low IL-10 producer haplotype (-1082*A, -819*T, -592*A) was significantly associated with HTLV-I carriage or HAM/TSP compared with healthy controls (*P=*0.02 and 0.01, respectively). Our results suggest that IL-10 -819*T and -592*A alleles are significant risk factors for developing HTLLV-I infection but do not appear to convey additional risk for developing HAM/TSP.

## Introduction

Human T cell lymphotropic virus type I (HTLV-I) infection is a causative factor of two diseases; namely HAM/TSP and adult T cell leukaemia (ATL) (1, 2). Endemic areas for HTLV-I infection include South-West Japan, parts of Africa and South America (3-6). More recently the North-East of Iran (Mash-had) has been also recognised as a HTLV-I endemic area (7). We have previously shown that the prevalence of HTLV-I infection in the Mashhad region is approximately 2.1% for the entire population and 0.44% in blood donors (7, 8). 

The mechanisms underlying the etiopathogenesis of (HTLV-I) associated myelopathy/tropical spastic paraparesis (HAM/TSP) in HTLV-I infected individuals are poorly understood. Viral factors and host genetic background both may influence the outcome of HAM/TSP in HTLV-I infection. The type of HTLV-I virus in Mashhad is the same as that found in Japan (7). However, there is no clear evidence of association between HTLV-I variants and susceptibility to HAM/TSP (9). While it has been suggested that an amino acid substitution in the Tax protein, which is essential for viral transcription, increases the risk of developing HAM/TSP after HTLV-I infection, mutation in the Tax gene is linked to HTLV-I subtype rather than risk of HAM/TSP (10, 11). Though, in one study, it was reported that a variant of the Tax gene was more frequently observed in patients with HAM/TSP compared with HTLV-I carriers (12). 

It is possible that infection with HTLV-I alone is not sufficient to cause HAM/TSP and that differences in the genetic background of individuals may be crucial for determining the progression of HTLV-I pathogenesis. A number of possible candidate genes have been implicated in HTLV-I infection. Human leukocyte antigen (HLA) class I and II genes have been carefully investigated in Japanese cases (13-17). 

Gene polymorphisms, which affect the inter-individual *in vitro* production of cytokines have been proposed as candidates for many infectious and non-infectious diseases. IL- 10 shows a variety of immunoregulatory properties, suggesting it is an important factor for maintaining immune and inflammatory responses. It inhibits the production of pro-inflammatory cytokines such as IL-1, IL-6 and TNF-α (18). The promoter region of IL-10 contains single nucleotide polymorphisms at positions -1082*G/A, -819*C/T and -592*C/A. The -819 and -592 alleles are so strongly linked that only three of the eight possible haplotypes segregate in Caucasian populations, namely GCC, ACC and ATA for alleles at -1082, -819 and -592, respectively (19, 20). The –1082*G allele is associated with higher IL-10 production, while the –1082*A allele correlates with lower IL-10 production. The -819*T and –592*A alleles are associated with diminished IL-10 production. 

IL-10 promoter polymorphisms with outcomes of viral infections such as HIV and HBV have been reported (21, 22). Recently, it has been shown that the IL-10 -592*A allele is associated with lower HTLV-I Tax-induced transcription activity and risk of HAM/TSP in Japanese HTLV-I patients (23). In the present study we investigated the association between IL-10 polymorphisms and the outcome of HTLV-I infection in Iranian patients. 

## Material and Methods

*Collection of samples*

All participants in this study were Iranians sharing the same ethnic background and residing in the Khorasan province. They included 177 healthy controls, 80 randomly selected HTLV-I carriers and 75 patients who fulfilled the accepted criteria for HAM/TSP (24). The average age of HAM/TSP patients was 47±9.8 years (range 27-76), and the female to male ratio was 1:5. The clinical and laboratory findings related to these patients have been described previously (25). Blood samples from HTLV-I carriers and healthy controls were obtained from individuals attending the Mashhad blood transfusion centre. The average age of the carriers was 37.9±10.4 years, and 25 of the 80 carriers were women (37.5%). The average age of the controls was 34.2± 8.4 years. The study protocol was approved by the Ethics Committee of the Ghaem Hospital, Mashhad University of Medical Sciences and Mashhad blood transfusion centre. All of the cases and healthy controls gave informed consent to participate in this study.

*IL-10 genotyping*

DNA was extracted from 5 ml EDTA whole blood samples using a standard phenol-chloroform method. The ARMS-PCR technique was used to genotype individual’s samples for IL-10 polymorphisms at positions -1082*G/A, -819*C/T and -592*C/A using allele-specific primers as previously described (26). 

1.5 μ of DNA (100-250 ng/ μl) was amplified in a 10 μl master mix solution containing 1.25 U Thermoprime Plus DNA polymerase, 75 mM Tris-HCl, 20 mM (NH_4_)_2_SO_4_, 2.0 mM MgCl_2_, 0.01% Tween 20, and 0.2 mM of each of dATP, dCTP, dGTP, and DTT (all ABgene, Epsom, United Kingdom). Growth hormone primers were used as an internal control (10μl of each). A volume of 5.5 μl of DNA plus master mix reaction was aliquoted into microtiter plate wells containing 5 μl of one or the other specific primer mixes (10 μM each). The protocol for the PTC-100 PCR machine (MJ Research) was as follows: 1 minute at 96°C followed by 10 cycles of 15 secs at 95°C, 50 secs at 65°C, 40 secs at 72°C followed by 20 cycles of 20 secs at 95°C, 50 secs at 59°C and 50 secs at 72°C. The amplified products were visualized on a 2% agarose gel against 200 bp DNA ladders and stained with 5 μl (0.5 μg/ml) of ethidium bromide.

*Statistic analysis*

Differences in IL-10 genotype, allele and haplotype frequencies between HAM/TSP patients, HTLV-I carriers and healthy controls were performed using STATA version 8 (STATA Corp,). The significance of differences in allele and haplotype frequencies between HAM/TSP patients and HTLV-I carriers, HAM/TSP patients and healthy controls and HTLV-I carriers and healthy controls were established using the Fisher exact test and by calculation of odds ratios (OR) with 95% confidence intervals (95% CI). The significance of genotype frequency differences between any of the three groups was determined by the Chi-squared test.

## Results

The IL-10 -819*T allele is associated with HTLV-I carriage and HAM/TSP

The distribution of IL-10 -819*C/T allele and genotype frequencies in HAM/TSP patients, HTLV-I carriers and healthy controls were also examined. The frequency of the low IL-10 producer -819*T allele was higher in HAM/TSP patients (38%) and carriers (36.9%) compared with healthy controls (26.8%) (Table 1). A significantly higher frequency of the T allele was observed in HAM/TSP patients compared with healthy controls (*P=*0.01; OR=1.67 (1.08-2.55) and in carriers compared with healthy controls (*P=*0.02; OR=1.59 (1.04-2.44). There was no significant difference between carriers and HAM/TSP patients (*P=*0.91; OR=0.91). Furthermore, the frequency of the homozygous lower producer -819*T/T genotype was greater in the HAM/TSP patients (13.4%, *P=*0.04) and carriers (16.3%, *P=*0.05) than in controls (7.3%). 

**Table 1:** Allele and genotype frequency of IL-10 polymorphisms

Position of IL-10 polymorphisms	Controls Total=177 n (%)	Carriers Total=80 n (%)	HAM/TSP Total =75 n (%)
IL-10 -1082*G/A			
Allele G A	121 (34.2) 233 (65.8)	49 (30.6) 111 (69.4)	47 (31.3) 103 (68.7)
Genotype			
GG AG AA	15 (8.5) 91 (51.4) 71 (40.1)	5 (6.2) 39 (48.8) 36 (45.0)	8 (10.7) 31 (41.3) 36 (48.0)
IL-10 –819*C/T			
Allele C T	259 (73.2) 95 (26.8)	101 (63.1) 59 (36.9)^a^	93 (62.0) 57 (38.0)^b^
Genotype			
CC CT TT	95 (53.7) 69 (39.0) 13 (7.3)	34 (42.2) 33 (41.2) 13 (16.3)^c^	28 (37.3) 37 (49.3) 10 (13.4)^d^
IL-10 -592*C/A			
Allele C A	259 (73.2) 95 (26.8)	101 (63.1) 59 (36.9)a	93 (62.0) 57 (38.0)b
Genotype			
CC AC AA	95 (53.7) 69 (39.0) 13 (7. 3)	34 (42.2) 33 (41.2) 13 (16.3)c	28 (37.3) 37 (49.3) 10 (13.4)d

^a^ *P* =0.02 vs. controls

^b^ *P* =0.01 vs. controls.

^c^ *P* =0.04 vs. controls.

^d^ *P* =0.04 vs. controls.

The IL-10 -592*A allele is associated with HTLV-I carriage and HAM/TSP

Because the IL-10 -819*C/T and the -592*C/A polymorphisms are very strongly linked, the result from the analysis of the -592 polymorphisms is identical to that described above. 

To determine whether an association exists between HTLV-I infection, *per se*, and the IL-10 -819*C/T and -592*C/A polymorphisms, the genotypes of the HAM/TSP patients and HTLV-I carriers were combined. A highly significant association was identified in the genotype and allele frequencies compared with controls (*P=*0.01 and *P=*0.004 respectively, data not shown).

*The IL-10 -1082 alleles are not associated with HTLV-I infection or HAM/TSP*

Allele and genotype frequencies of IL-10 gene polymorphisms (-1082*G/A, -819*C/T and -592*C/A) were determined in patients with HAM/TSP, healthy controls and HTLV-I carriers. The frequency of the high IL-10 producer -1082*G allele was the same in all three groups and no significant differences were observed between HAM/TSP and healthy controls, carriers and controls or between HAM/TSP and HTLV-I carriers (Table 1).

*The IL-10 haplotype ‘ATA’ is associated with HTLV-I carriage and HAM/TSP*

In this study we also compared the frequencies of the three common GCC, ACC and ATA IL-10 haplotypes in HAM/TSP patients, carriers and controls (Table 2). There were no significant differences in GCC and ACC haplotype frequencies between any of the groups. By contrast, the low producer ATA haplotype was present at higher frequencies in HTLV-I carriers (36.9%, *P=*0.02; OR= 1.59 (1.04-2.41) and in patients with HAM/TSP (37.3%, *P=*0.02; OR= 1.62 (1.05-2.48) compared with controls (26.8%). For HTLV-I infected individuals (HAM/TSP and HTLV-I carriers combined) a significant difference in ATA haplotype frequencies was observed compared with controls (*P=*0.005; OR; 1.60 (1.14-2.26) (Table 2). 

## Discussion

*The possible influence of IL-10 genotype on HTLV-I infection*

IL-10 may influence HTLV-I infection in several ways (a) because it has a variety of immunoregulatory functions and (b) because it is a differentiation factor for virus-specific cytotoxic T cells. For example, IL-10 inhibits CD80/CD86 expression by antigen presenting cells (APC), reducing the capacity of these cells to provide the co-stimulatory signals essential for T cell activation and cytokine production (27). Thus, too little IL-10 in a subset of HTLV-I infected individuals may allow inflammatory mechanisms that lead to HAM/TSP. Hence, one of the possible mechanisms involved in the outcome of HTLV-I infection may be related to the inhibitory effects of IL-10 on the production of inflammatory cytokines such as TNF-α, IL-1β and IL-6 which probably contribute to the pathogenesis of HAM/TSP.

**Table 2:** Distribution of IL-10 haplotypes in healthy controls, HAM/TSP patients and HTLV-I carriers

IL-10 haplotype	Controls total=354 n (%)	Carriers total=160 n (%)	HAM/TSP total=150 n (%)	HAM/TSPH and Carriers (total=310) n (%)
GCC	120 (33.9)	49 (30.6)	45 (30.0)	94 (30.3)
ACC	139 (39.3)	52 (32.5)	49 (32.7)	101 (32.6)
ATA^*^	95 (26.8)	59 (36.9)	56 (37.3)	115 (37.1)

^*^Significant differences were observed between HAM/TSP patients and healthy controls (*P=*0.02, OR; 1.62, 95% CI: 1.05-2.48), HTLV-I carriers and healthy controls (*P=*0.02, OR; 1.59, 95% CI: 1.04-2.41), and HTLV-I infected individuals with controls (*P=*0.005, OR; 1.60, 95% CI: 1.14-2.26).

In contrast, IL-10 acts as a T- cell differentiation factor, promoting the number of IL-2-activated cytotoxic T lymphocytes (CTL) (28). In the mouse, systemic administration of IL-10 leads to acceleration of cardiac allograft rejection and generation of donor-specific CTL (29), whereas allograft survival is prolonged in normal as well as presensitized recipients given anti-IL-10 antibody (30). IL-10 is able to induce proliferation and cytotoxic activity of papillomavirus- specific CD8+ CTL. It also up-regulates intercellular Th1 cytokine production and perforin accumulation in specific CTLs (31). In contrast, the up regulation of IL-10 in persistent viral infection such as lymphocytic chroriomeningitis virus (LCMV) leads to an impaired T cell response. Removal of IL-10 resulted in the maintenance of robust effectors T cell response and elimination of viral infection (32). However, it is not clear whether IL-10 expression, which is induced by HTLV-I tax compensates the effect of -819 and -592 polymorphisms in HTLV-I infection. 

Our hypothesis was that, because polymorphisms in the IL-10 promoter region are related to IL-10 production, the IL-10 genotype of HTLV-I infected individuals might influence the pathogenesis of HTLV-I infection. Our study group was restricted to patients from an area in North Eastern Iran.

*IL-10 genotype is associated with HTLV-I infection, but not with HAM/TSP development*

In this study we demonstrated that IL-10 -819*T and -592*A alleles were more frequent in HAM/TSP patients and HTLV-I carriers compared with healthy controls. Only the three anticipated IL-10 haplotypes, GCC, ACC and ATA were identified in the population from North East Iran. 

IL-10 genotype seemed to influence HTLV-I infection *per *se and not the development of HAM/TSP in a subset of infected patients. HTLV-I carriers and HAM/TSP patients were not significantly different for IL-10 allele or genotype frequency, although these HTLV-I infected groups, separately and collectively, were different from healthy, uninfected controls. These data therefore, suggest that low IL-10 production does not per se contribute to the development of HAM/TSP after HTLV-I infection. In a recent study (23) on Japanese patients, significant difference was observed in the distribution of the IL-10 -592*C/A polymorphism between HAM/TSP patients and asymptomatic carriers. This contradicts our observations of an Iranian and those reported from a Brazilain population (34). The explanation for this discrepancy is unclear but clearly genetic differences exist within the IL-10 gene between these populations. For example, no linkage disequilibrium (LD) is apparent between -592 and -819 SNP in the Japanese population whereas extremely strong LD exists in Iranians. It is not clear from the study of Sabouri *et al*., (23) whether both HTLV-infected carriers and HAM/TSP patients have increased -592A frequencies compared with controls with the highest frequency being seen in carriers; or whether -592A frequency in patients developing HAM/TSP are the same as in the normal population. However, it does suggest that the overall risk of susceptibility to HTLV-I infection is associated with the -592 position and not the -819. As much it helps disentangle these two SNP associations observed in the Iranian population, clearly further studies are required to categorically confirm whether -592A allele is protective or not for HAM/TSP development. Our data suggest that low IL-10 production does not contribute to the pathogenesis of HAM/TSP after HTLV-I infection.

*IL-10 genotype and viral infections*

IL-10 genotype and viral immunity have been studied for other infections. The IL-10 –819*T and –592*A alleles have now been linked to other viral infections such as hepatitis B virus (HBV) and Herpes Zoster (HZ) and human immunodeficiency virus (HIV) (21, 35, 36). The IL-10 ATA haplotype and the -819*T and -592*A alleles are more frequent in HBV carriers than patients with chronic progressive liver disease. In contrast, in patients with HZ, the frequency of the ATA haplotype is significantly increased compared with controls, which may suggest that low IL-10 production permits the virus to replicate more effectively in patients with HZ or HTLV-I. Furthermore, the -592*A allele is associated with accelerated disease progression of acquired immune deficiency syndrome (AIDS) **(**22). 

*Genotype-phenotype interpretation*

The identification of low IL-10 producer genotypes is based on *in vitro* evidence. It is a major assumption that the same association holds *in vivo* in HTLV-I infected patients. The serum levels of IL-10 in healthy controls, HAM/TSP patients and HTLV-I carriers are not known. This would have been informative in relation to IL-10 genotype and the role of IL-10 in HAM/TSP patients or HTLV-I carriers.

Another concern is that the -592*A/ -819*T alleles are associated with HTLV-I infection while the -1082 alleles are not. The -1082 polymorphism coincides with a binding site for ETS-1, a lymphocyte transcription factor, while the -592 polymorphism coincides with a PU-1 transcription factor expressed in cells of the monocyte lineage. Consequently, based on our findings, it may be that lymphocyte-derived IL-10 is not an important regulator of HTLV-I immunity, but macrophage or APC-derived IL-10 does influence responses to HTLV-I.

Although we do not have information about the viral load of these patients, low production of IL-10 in HAM/TSP patients and HTLV-I carriers could be responsible for insufficient induction of a CTL response and perforin production, which is necessary for elimination of HTLV-I infected target cells. However, it is necessary to take into account that low IL-10 production induced a T helper response and may contribute to the HTLV-I elimination. Although this mechanism has been suggested in other viral infections such HIV (36), the results should be carefully interpreted due to the lack of suitable animal model of HTLV-I infection.

## Conclusion

We conclude that the IL-10 -819*C/T and -592*C/A polymorphisms may contribute to susceptibility to HTLV-I infection in an Iranian population, although it would not appear to be related to any additional risk ad protection for developing HAM/TSP.
